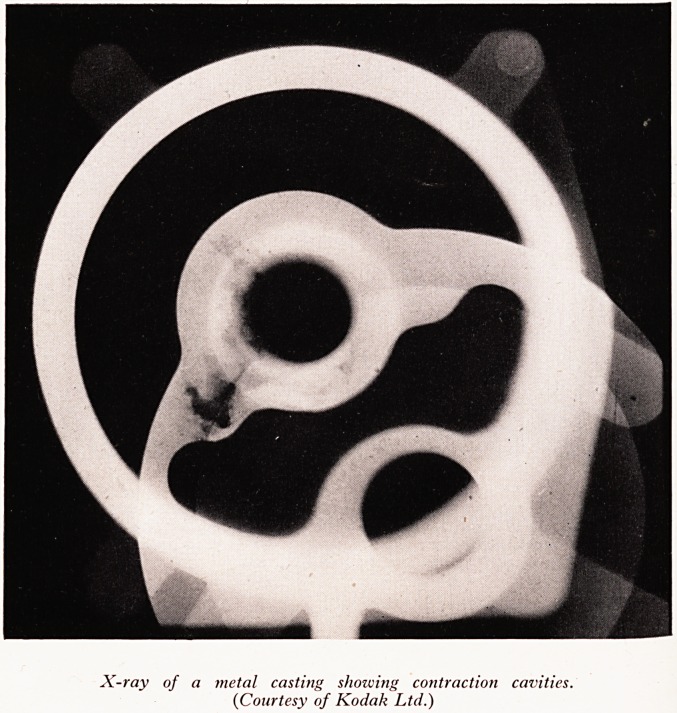# X-Rays in Everyday Life

**Published:** 1960-07

**Authors:** J. H. Middlemiss

**Affiliations:** Director of Radiology, United Bristol Hospital


### X-RAYS IN EVERYDAY LIFE

### LONG FOX MEMORIAL LECTURE—13th OCTOBER 1959

BY

J. H. MIDDLEMISS

*Director of Radiology, United Bristol Hospital*

Many of those who have had the honour of delivering this lecture were, or still are, distinguished men. I am privileged to find myself named among them, but this has made me acutely conscious of my own shortcomings. This is no mock modesty, for what can a radiologist discuss apart from technical aspects of his work, that is not well known to experts in other fields ? For X-rays are the handmaiden of experts in all scientific walks of life and lie within the experience of the man in the street.

There is however, one course open to me: to take a cross-section of life and to see how and where X-rays enter into the lives of us all. I think I may surprise some of you, I hope I may interest all.

X-rays are a form of energy. They are a form of electro-magnetic radiation, and part of the whole spectrum of electro-magnetic radiation, of which visible light is another part. At one extreme are wireless waves, towards the other extreme X-rays, wavelength being the fundamental difference between these various forms of radiation. Whereas wireless waves have a wavelength measured in metres, those of X-rays are measured in Ångstroms (which are units of length about the size of atoms).

X-rays have several properties: They can penetrate matter; they have biological effects; they have a photographic effect, and can be diffracted. We have made use of all these properties; one—the biological effect—has inherent dangers, and I shall discuss these at some length later.

In the modern community life which has evolved in the Western part of this planet, some of the scientific aspects of which are now being applied by other communities, X-rays have come to play an integral part. They are part of the whole, part of our culture. Advances in civilized life are achieved in stages: research is undertaken; some fruits of this are applicable to everyday life; those that are useful are developed and then may influence our way of life. Often it is only the end that is apparent, the means to the end is not evident, so it is that the means to the end becomes accepted and incorporated in our life without the community at large realizing this.

### APPLICATION OF X-RAYS IN MEDICINE

*Use of the Biological Effects.* In the field of medicine use has been made of the biological effect for the treatment of patients. The effect of X-rays on living tissues is to cause an increased blood-flow to those tissues and then with higher doses to cause the death of the tissues. At one time it was hoped that this might be a universal cure for cancer and extravagant claims were made for it. These claims were not all substantiated and as a result the method fell for a time into disfavour. Fortunately there has been a resurgence and it is a fact that many people to-day owe their lives to it, for in selected cases and certain types of cancer it is the treatment of choice with remarkable results, apparent complete cure being procured. In many other cases the final outcome is hopeless but palliation can be obtained and months and even years of comfort and useful life achieved.

This form of treatment is also an accepted regime for certain types of arthritis and rheumatism. In skilled hands remarkably good results can be obtained in many instances. It is not a cure, but it can relieve pain, and may sometimes halt progression of the disease.

Again for certain types of skin disease this is the treatment of choice, cure often being obtained regularly by this method where other methods fail.

A newer application of the biological effects of X-rays is to sterilize medical equipment and vaccines, and to disinfect packaged products and foods. Bacterial and virus contamination can be eliminated by irradiation. The method is already being used, and at the present time a new plant is being built at the Wantage Radiation Laboratories under the direction of the Atomic Energy Research Establishment, for testing the process on a large scale—it is hoped there to render sterile three tons of equipment a day.

*X-rays in Diagnosis.* Next, in the field of medicine, the property of X-rays to penetrate matter and produce a photographic effect has been put to use. This is the application with which most people are familiar—the field of medical diagnosis—my own specialty. It is now a vast, complicated and expensive science. It can be classified mainly into four categories, prophylactic, diagnostic, control of treatment, and research.

The Prophylactic use includes the detection and prevention of spread of disease. It was early employed for occupational diseases such as coalminer's pneumoconiosis and silicosis. More recently—in the post-war period—pulmonary tuberculosis has been virtually eliminated. This has been due to the triad—early radiological diagnosis, new effective anti-tuberculous drugs and an increase in the general standard of nutrition and housing of the nation.

In practical medicine, firm and definite diagnosis is an essential pre-requisite to proper treatment. Under many conditions, radiology has its part to play in diagnosis. During the years of development of this science, there have been periods of abuse, when it has been regarded as the be-all and end-all of diagnosis. We have fortunately come through that phase and radiology is now taking its place in proper perspective—as a useful diagnostic aid in the diagnosis of many ailments, alongside other tools which aid the art of the doctor—clinical acumen, experience, laboratory methods and so forth. In its proper place and used logically, it is invaluable; but what experienced doctors realize, though this is unfortunately not yet realized widely by the laity, is that X-rays have their limitations and pitfalls.

In the control of treatment the great benefits of radiodiagnosis are well illustrated in that common misfortune—fracture of the femur in the elderly. Without X-rays a patient might have to spend many months in bed; but with proper radiological facilities it is now common practice to nail the hip so that within a few weeks the patient can be back home and doing her shopping. In medical research the use of radiological methods seems endless. Usually the X-ray method is not the whole, but is merely part of a team approach to some problem. Thus, in the rapid strides that have been made in the surgery of the heart, radiological investigation has been an essential pre-operative measure to establish a diagnosis and so influence surgical approach and treatment.

### VETERINARY USES

In veterinary work X-rays are being used increasingly both as a diagnostic tool and as a research method. As a diagnostic tool they can demonstrate anything from urinary stones to confirmation of pregnancy, from lung diseases to fractures. The animal examined may be anything from an elephant to a bat. At the London Zoo now there is a treatment centre with a well-equipped operating theatre, provided with full X-ray facilities (see Plate VIII).

As examples of research methods X-rays have been used to investigate the gastro-intestinal tract of ruminants and recently an entire book has been devoted to this subject. Similarly X-rays are now in use to investigate the cause of and treatment of rickets in South American monkeys. Some Scottish sheep do not thrive and do not produce live young when grazed on certain lowland pastures. By studying the delay in ossification and the degree of mineralization of bones of these sheep, it has been possible to incriminate the mineral content of the soil on which the pasture was growing. In some cases it has been possible to rectify this, and to show improved mineralization of the sheep's bones.

### TESTING WORKS OF ART

In non-destructive testing of antiquities and works of art, X-rays have a valuable place. For example, at the British Museum there is a laboratory for such testing and X-ray apparatus is amongst its tools. There the work done consists of the examination and identification of such objects as bronzes, iron, marble and ceramics and the examination of these for flaws, cracks, repairs, artefacts, inlays, and for determining the way they were made. There I have seen jet beads being examined to determine the direction of their holes, bronze figures to see if they are in one piece or whether the head has been joined subsequently; articles that cannot be opened (e.g. a mummy) examined to see what was inside (see Plate IX), pots to see if they have been broken and later repaired, bronze sword handles to see their method of attachment, and clay tablets for identification of inscriptions. What is demonstrated by this method may contribute to knowledge of structure, and often influences the decision to purchase or the price to be paid if flaws are demonstrated. In the realm of conservation most objects, before restoration or routine maintenance is undertaken, are examined by this method.

At the National Gallery, Dr. Rawlins, the Physicist-in-charge of the Research Laboratory has devized for non-destructive testing of paintings an X-ray method based on the differential X-ray absorption of the various thicknesses of the different pigments. Discrimination between old and modern paintings is possible because lead pigments were used in the older paintings, whereas more recent oil colours are less opaque to X-rays. In addition, modern priming is more opaque than ancient priming and accordingly the radiograph of a modern painting reveals no features of the visual image other than the highlights. X-rays thus reveal much of the artist's technique, the nature of the pigments he used, and details of his brush work. In this way old masters of the South European School, can be differentiated from those of the North European School. Both groups painted on wood panels, which was usually poplar, and they primed these with a covering of gypsum on which they drew in chalk, then painted. Rafael, an example of the Southern European School, would paint the surround heavily with lead paints, but only the highlights of his figure, leaving the flesh figure in gypsum. An X-ray of this shows an opaque surround and a non-opaque figure. Rubens, on the other hand, an example of the North European School, painted the figure heavily with lead paints, but the surround he painted less heavily with lakes and other less dense paints. An X-ray of this shows the reverse of the other. In this way also the alterations by the original artist, modifications by later painters and forgeries can be detected.

The method is also used routinely before restoration to show cracks, breaks, and blisters; once these have been demonstrated they can be readily repaired.

### DETECTION OF CRIME

In forensic work and criminology this tool is again useful. For instance, all mail entering scheduled establishments, e.g. Harwell, is screened fluoroscopically. In Tanganyika, I have seen all workers leaving the Williamson diamond mines each day being screened fluoroscopically, to see whether or not they are carrying away diamonds. Localization of foreign bodies is of course a simple procedure, much the same technically whether it is localizing a bullet in a corpse, or say, a swallowed hair grip in a child, which is a daily event in my department. With the police in Bristol, we have been trying to devise a way of X-raying the fingerprints of those corpses from whom the outer skin surface has been eroded; for when this occurs it is impossible to obtain finger prints by normal photographic methods. Similarly, it is possible to examine postage stamps by the use of very soft X-rays, even to see the watermark and to detect forgeries in this way.

### X-RAYS IN INDUSTRY

First let us consider welding. Two metal surfaces are joined by a process, usually with the use of an oxy-acetylene arc, which melts the two adjacent surfaces and a third metallic source, and all three are allowed to intermix. Then on cooling this fuses together. If the technique of the craftsman is at fault, flaws may arise at the junction giving a joint which is insecure and may even be dangerous. Inspection of welds by radiography is now normal practice in many industries and in some it is compulsory.

In 1930 the United States Navy decided to accept fusion-welded steam vessels provided that radiographic technique became an accepted inspection procedure for these welds. In 1934, Lloyds Register of Shipping wrote this inspection technique into new codes for the acceptance of welded vessels. And now radiography is widely used for inspecting large welded structures. The type of defects that may be detected are blowholes, cracks (see Plate X), inclusions, large grain size, burnt metal, locked up stresses, porosity (see Plate XI), and so forth. Moreover, X-ray inspection is of great value in the training of welders and their attainment of approved standards. Insurance companies whose inspectors supervise every step of such procedures in many cases insist on their approval of both the X-ray department and the industrial radiologist.

This technique is now applied to all pressure containers (e.g. ships' boilers, gas-holders, steam generating plants, chemical reaction equipment, chemical containers such as those for butane or ammonia for refrigeraters, boilers for power stations, paint kettles for paint manufacturers); it is applied to oil pipe lines, ship-building, atomic power stations, railway engine construction, aircraft production, and in the constructional trade by the big contractors.

Next let us turn to casting. A casting is a piece of metal shaped in a mould which is a pattern of a shaped cavity prepared to receive molten metal. There are various methods of casting, such as, sand casting, die casting, centrifugal casting, precision casting, and shell moulding. They may be enormous as are some of the steel castings or they may be light alloy castings. Radiography is a routine method of inspection of these foundry products. It may reveal inhomogeneity in what is outwardly solid metal. This inhomogeneity may be due to contraction cavities (see Plate XII), gas evolved by the metal during contraction, inclusions, or interaction between the mould material and the liquid metal. Defects thus shown have influenced foundry technique considerably, thus shrinkage during cooling must be overcome by what is called "topping up"—the exact method of this topping up depends on the cooling curve of the metal concerned and the shape being produced.

X-ray inspection of castings for machine parts is widely used in the aircraft industry, and it is used extensively in the steel industry for inspection of tungsten steel, of die casts for printing presses, of accumulator grids and of machine tools. In any factory in this country where there is precision machinery, much of that machinery has been cast and much of it has been X-rayed during production.

In the aircraft industry, in addition to the welds and castings already mentioned, X-ray inspection of construction techniques and stress points is common practice. This is carried out both during construction and also after a specified number of flying hours (10,000) or a specified number of landings (500). What happens is this. During construction and testing, stress experts work out which spars and struts and components require examination. The radiology department then X-rays the parts concerned working out a technique for the examination of each part—not an easy problem when heavy X-ray apparatus has to be manipulated around an aircraft. For some examinations the apparatus has to be manhandled into the craft, whereas others

### PLATE VIII

K-*ray of a Tiger's fore-foot showing osteomyelitis of the radius. (Taken at the London Zoo—courtesy of Mr. Graham Jones.)*

*A mummified bird removed from a tomb shown by X-ray examination without removing the outer casing.* (*Courtesy of Kodak Ltd.*)

PLATE X

*X-ray of the emergency exit of an aircraft where two metal surfaces have been welded together showing cracks and blowholes. These defects are not acceptable.* (*Courtesy of B.A.C. Ltd.*)

PLATE XI

*X-ray of a weld showing porosity. This result is not acceptable. (Courtesy of B.A.C. Ltd.)*

PLATE XII

*X-ray of a metal casting showing contraction cavities. (Courtesy of Kodak Ltd.)*

can be carried out from outside. Wings and body-work and undercarriages are examined at selected points. The technique for the examination of all these parts is then written into the instructions that go with the aircraft to the buyer. The faults that are detected in this way are cracks and fractures—metal fatigue. Some of these defects may call for the replacing of a spar, while some are merely splinted by rivetting metal plates across them.

There are other parts of aircraft which need inspecting radiographically when the plane is in commission. These include rivettings to see that the outer fabric is correctly rivetted to the spars, electric wires in propellers for the de-icing apparatus, and oil coolers after cleaning to see that all contaminants have been removed, for if all sludge and metallic foreign bodies are not removed engine failure may develop.

In industry, such as manufacture of rubber products radiological inspection of mouldings is widely used. For example, radiography is an essential tool in the manufacture of motor tyres. The rubber is moulded onto a fabric base. For research purposes fine wires are embedded in selected places in a tyre during manufacture, in order to follow any changes in structure during running. Individual cords in the fabric, and the continuity of them, are investigated by injecting liquid mercury which flows along the cords, and the tyre is then X-rayed. This technique is also used in the rubber industry to inspect golf balls, belting and hose.

Nowadays inspection of assembled objects by radiography is standard practice. Examples of this are wireless valves to determine correct positioning of grids, electric light bulbs, X-ray tubes (it seems rather paradoxical to X-ray an X-ray tube) and sealed hydraulic cylinders to isolate faults.

In the textile industry, cloths are inspected by radiography; and likewise it is possible to determine whether pearls are cultured or are natural.

Inspection by fluoroscopy is used on an even wider front. Fluoroscopy is the technique whereby X-rays are directed through the object being examined onto a fluorescent screen which an observer watches. The objects are usually put on a moving belt and move across in front of the screen giving the observer time to inspect for defects or foreign bodies and time to pick out any that he wishes to examine more fully. No permanent record, such as a photograph, is made in this case. This technique is used for inspecting packaged razor blades to determine whether any have been damaged by the packing machinery; for ascertaining the nature of glass used in the manufacture of clinical thermometers, to determine the accurate positioning of inserts in insulators, to detect the presence of foreign bodies such as glass in synthetic sponges, to eliminate faults and calcareous plaques from skins, particularly crocodile skins, before tanning, and to ensure centralization of the core and also bonding of joints in cable manufacture and so on. Fluoroscopic screening is also used in the food and confectionery industries. I have visited chocolate and sweet factories where anything from 2,000 to 5,000 boxes or packages are inspected a day, and where perhaps forty or fifty packages a day are rejected by the observer; these packages are opened and in fifteen or twenty, a metallic fragment is recovered, perhaps a chip from the machinery or a nail from a crate. Some bakeries use this method to detect opaque foreign bodies which may have been included with the dried fruit. Baby foods, sausages and pies, tinned or packaged foods and frozen vegetables are other everyday commodities examined by fluoroscopy. Many materials are screened in bulk before being tinned or used for manufacture, like peanuts, to eliminate stones. Some importers now inspect citrous fruits (oranges and grape-fruits) for crystallization, frost damage and puffiness in order to reject the bad fruit.

There seems to be no end to the ingenious ways in which X-rays may be used in industry. Viscosity of opaque fluids has recently been measured by X-raying a metal ball dropping through the fluid. The firm concerned was manufacturing ink for ball-point pens, and the viscosity and consistency of the ink are critical. This method of inspection solved their problem.

*Flash radiography* is another technique which is used to examine very rapidly moving objects by means of a single intense pulse of X-rays. Objects can be studied when they are beyond the reach of high speed photography with visual light, for example in explosions when the flight of projectiles is obscured by smoke and flame.

*Micro-radiography* is the taking of a radiograph with very soft X-rays through very thin sections. It can be used for biological specimens, paper, fabrics, or metals. Organic slices can be cut with a microtome; metallic slices can be cut at about one sixteenth of an inch thickness and then polished down further. The resultant radiograph can be magnified from 200- to 500-fold for study. This technique can be used to tackle such problems as the segregation of constituents in alloys, the distribution of elements in steel and the effect of any such segregation on machinability.

### X-RAY DIFFRACTION

In 1912 von Laue discovered that the regular network of atoms in a crystalline substance provides a three-dimensional grating suitable for the diffraction of X-rays. The interaction of X-rays with the atomic pattern of a crystal depends on the similarity between the wavelength of the X-rays and the regular distances between the scattering centres in the atomic pattern. Inter-atomic distances are about one Ångstrom, so X-rays of about one Ångstrom wavelength will be diffracted at angles convenient for registration on a photographic film. From this, atomic arrangements can be calculated, and in this way our knowledge of atomic structure has been enormously expanded. X-rays have, by this method, disclosed the underlying atomic patterns not only of such recognizably crystalline substances as quartz and diamond, but also of metals and alloys, inorganic chemicals such as the various minerals in the earth's crust and of natural organic fibres such as silk and cotton and synthetic polymers like nylon and polythene.

From its earliest days it was apparent that X-ray diffraction was an analytical tool of great value which could be used to supplement physical and chemical methods. Now it has developed into a large science, with even the formation of an International Union of X-ray Crystallography.

The applications of this procedure which affect our everyday life are diverse. For example, impurities in petrol are analyzed thus by the petrol firms; the various crystal forms of silica can be differentiated for the manufacture of steel and glass; the crystal form of magnesium tungstate required for fluorescent lamps is critical and is identified by this method; in metallurgy the orientation of grains and crystallites in ingots have considerable bearing on the properties of that particular metal, as for instance the power of electro-deposited chromium to remain bright; the tensile strength of nylon may be increased very considerably by cold stretching which has the effect of producing a directionally orientated arrangement of molecules with respect to the fibre axis, all of which has been investigated and subsequently controlled by this method; similarly gloss and sheen may be affected by the molecular orientation, as shown by X-ray diffraction patterns.

### ECONOMICS

*The cost* of all this is difficult to assess with any degree of accuracy. From figures made available by the Ministry of Health I have estimated that in the financial year 1957–8, the use of X-rays in the Health Service cost about £22,000,000. The cost in industry must be considerably less than that figure, but it is even more difficult to estimate. In the Health Service there are employed approximately 1,000 radiologists (or radiologists in training), 3,500 radiographers, 2,500 clerical and dark-room workers —a total of around 7,000. I have been unable to find a means of estimating accurately the number employed on this work in industry. The rate of work in some industrial establishments is greater than in medicine; for example in my department in the United Bristol Hospitals, a large busy teaching hospital, carrying out 70,000 X-ray examinations a year, we process an average of 600 films a day. At the Rolls Royce Works in Derby which I have visited, the X-ray department, X-raying component parts of aero jet engines, processes 1,600 films a day and has an annual film bill of £48,000. There are about six or seven firms in the country (for example Stirling Metals and Kent Alloys) that each process over 2,000 films a day.

I understand from the film manufacturers that about 30 per cent of their production goes to industry and 70 per cent to medical uses, but this is not a strictly accurate yard-stick because of course many more of the smaller films are used in industry than in medicine, and cost is related to area of film used and not to the number of them. However, taking into consideration all these factors I estimate that there are about 2,000 workers on the industrial side of X-ray work, and that the cost to industry of the work is of the order of £7,000,000 per annum.

There is one other aspect to be considered here—the organization and number of people concerned with producing X-ray apparatus and films. Most of the firms manufacturing X-ray equipment are now subsidiaries of larger concerns and so again it is not possible to arrive at any exact figure of the number of people employed in the industry. The X-ray manufacturing side of these companies is not a thriving industry. Many thousands of pounds are poured into research and most firms just manage to remain solvent. If they had not the backing of their parent companies—for example, on the continent, Philips Ltd. of Eindhoven manufacture X-ray equipment but the vast safe umbrella of the financially successful electric lamp manufacturing company is there to shelter them—if they had not this backing it is unlikely that design and radiological advances would have moved forward at the pace that they have done. The film manufacturers, on the other hand, who are also the manufacturers of conventional photographic films, make their major profits from the sale of X-ray film and employ the major part of their staffs on its production. My assessment of the number of persons employed in the manufacture of X-ray apparatus, of X-ray tubes and other accessories, of X-ray film, of distribution and installation and maintenance in Great Britain is slightly under 10,000. Together with the figures I have already given, that means that probably somewhere in the region of 20,000 persons are employed one way or another in the realm of X-ray work, and that the cost transferred to the population either by the Exchequer or by industry is somewhere in the region of £29,000,000 or £30,000,000 per annum.

I do want to consider briefly the *saving* and *advantages* that this has brought to our way of life. Many of them are intangibles. Some are hard business facts. In medicine, health preserved, health restored are not factors that can be measured. Reduced patient/days in hospital mean hospital beds available for more people. Earlier return to work means less dislocation of that work and reduced domestic upset if wages are involved. These facts are all of social significance. To all this X-rays have contributed.

In industry costly procedures are not undertaken if they do not bear fruit. The early detection of defects in design cuts down production costs and increases productivity. Another factor here is the maintenance of safety levels in such things as high pressure boilers, aircraft, and railway engines. Government contracts for aircraft insist on a very high level of X-ray inspection throughout and quite apart from moral issues or business grounds, or Government demands, the insurance companies have realized that there is an actuarial significance not to be ignored. Again to all this progress X-rays have contributed much.

### DANGERS OF X-RAYS

R. W. Emerson in his essay on the Law of Compensation expounded that for every peak there is a corresponding trough and for every good a corresponding bad. X-rays, which have their contribution to make to our way of life, are not without a compensatory bad. X-rays are dangerous to life. This has been learned the hard way. Men have been maimed and have even lost their lives due to exposure to X-rays. We believe now that we know something of the extent of the danger and how to prevent these effects. Briefly there are three types of harmful effects which may arise: These are direct injury, such as burns or necrosis; secondly as a late result of excessive dose, either at one time or spread over a period, cancer may be caused; the commonest form of this so to be caused is leukaemia. Thirdly genetic mutations may be caused in the germ cells in the reproductive organs; these will not affect the individual concerned, but may manifest themselves in subsequent generations, in the progeny of the exposed individual.

The Medical Research Council has published in the last few years a series of reports showing what it considers to be safe limits of ionizing radiations, of which X-rays are one form. The basic principle is that nobody should ever be irradiated unnecessarily, but that provided certain precautions are observed, and provided there are proper indications for the exposure, there is a range of safety within which to work and within which the advantages that may accrue to the individual far outweigh the possible dangers. As a single example of this, let me tell you the significance of the dose received during X-ray examinations of the chest. It is considered that during life no person should receive a total body dose greater than 200r (the unit of measurement)—remembering that dosage does not wear off, it is accumulated by the individual; it is also considered that no individual should receive a dose to the reproductive organs greater than 50r during reproductive life. In a chest X-ray examination the individual receives a skin dose (and remember this is not a total body dose) of about 1r (and his permissible dose is 200r total body); and at the same time he receives by scattered rays a dose to the reproductive organs of about 0·1mr (i.e. a tenth of a thousandth of 1r, when his permissible dose is 50r). I think those figures put the matter into proper perspective.

### SUMMARY

I have endeavoured to show where X-rays impinge on our daily lives. In this country from the time we get up in the morning until we go to bed at night, there are very few aspects of our lives which have not been influenced by X-rays. The electricity supply for your razor, the printing press for your morning paper, the tyres on the car or bus that takes you to work and the petrol that drives it along, the nylon for your dress or your shirt, chromium in your towel rail, the aircraft that brings your overseas mail, the construction of your telephone, the ink in your pen, the sausage you have for lunch or the chocolate you eat afterwards, the fluorescent lighting in your office, criminal investigations in the community, the cost of the articles you buy, the taxes you pay, your health, possibly your death, the components of your refrigerator, the paint on your house, the pearls you gave your wife, the sponge you wash yourself with—all this and much more may be influenced in twentieth century Great Britain by X-rays. Now in the few minutes left to me I want to revert to the past. All that I have told you tonight stems from the discovery of X-rays by Professor William Conrad Rontgen, Professor of Physics in Wurzburg in Germany on 8th November 1895 (64 years ago). In the following seven weeks by intense effort and work he investigated the properties of these rays remarkably fully. He was a scientist and was interested only in the academic investigation of the phenomenon he had discovered, not in its application. In the course of his investigations he discovered the photographic properties of X-rays and among the photographs that he personally made there were two—Mrs. Rontgen's hand and his shot gun—anticipating curiously the applications, medical and industrial, that I have described to you tonight and which are now used so extensively. He published his findings on the 1st January 1896.

This annual lecture is in memory of Dr. Edward Long Fox, citizen of Bristol and respected physician. He was born in 1832 and practised medicine in this city until the time of his death in 1902. Professor Rontgen's discovery of X-rays was put to immediate use in medicine, and by the end of 1896 was an accepted if then rather dangerous tool. The last six years of Dr. Long Fox's life was thus in the period of early development of X-rays. In the medical records of Bristol which I have searched there are many references to their use. But in a thorough search of the records of Dr. Long Fox, I can find no reference to his using them.

## Figures and Tables

**Figure f1:**
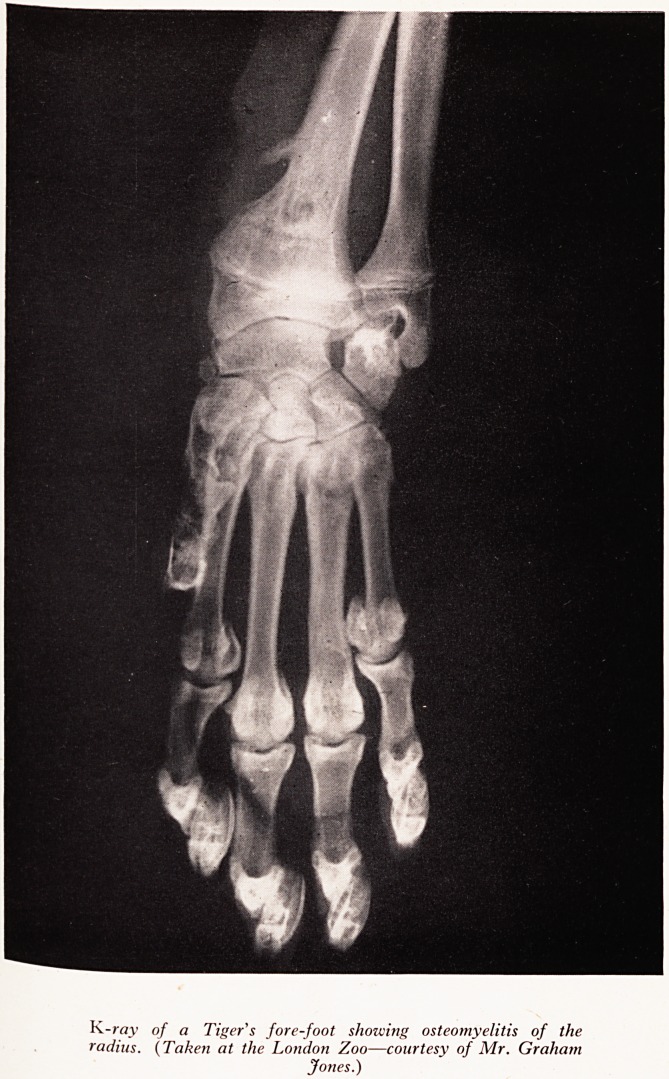


**Figure f2:**
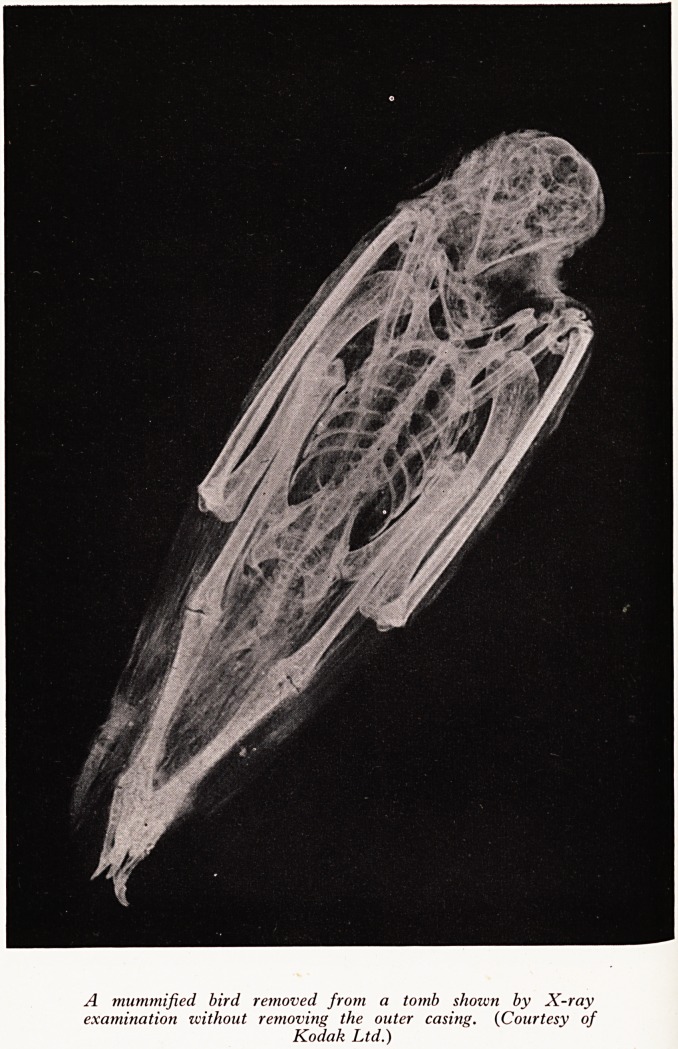


**Figure f3:**
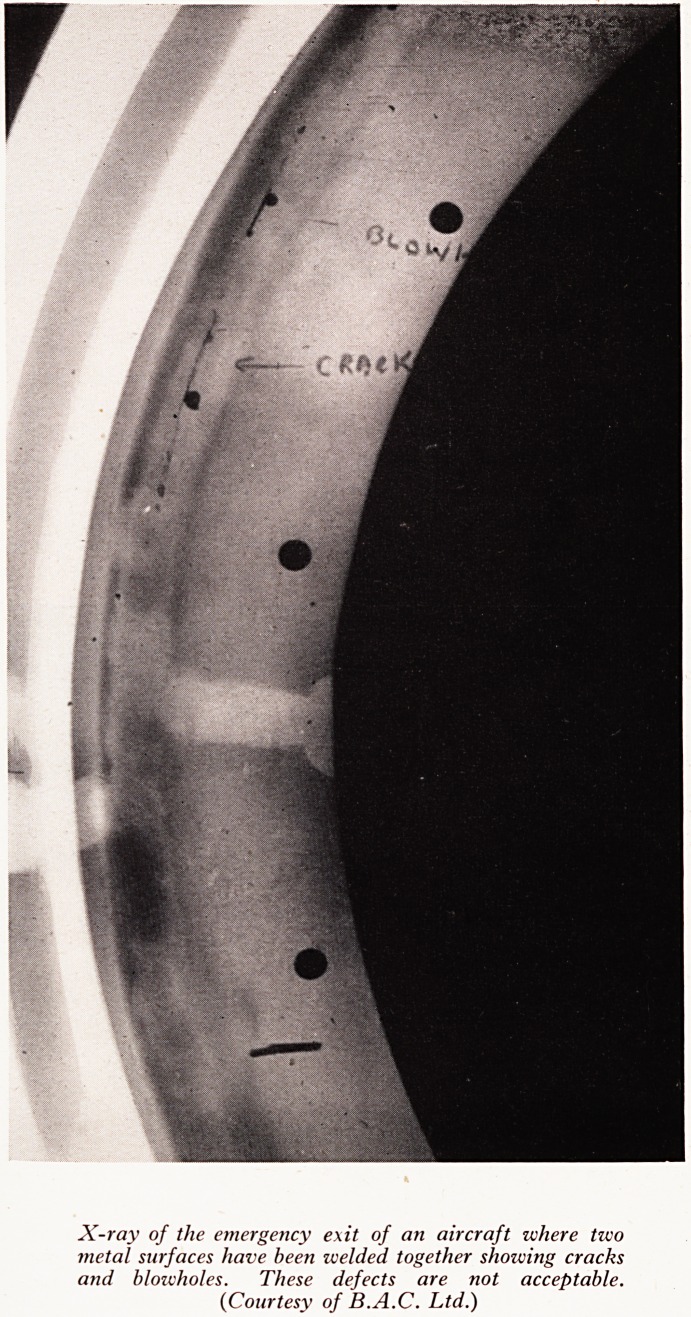


**Figure f4:**
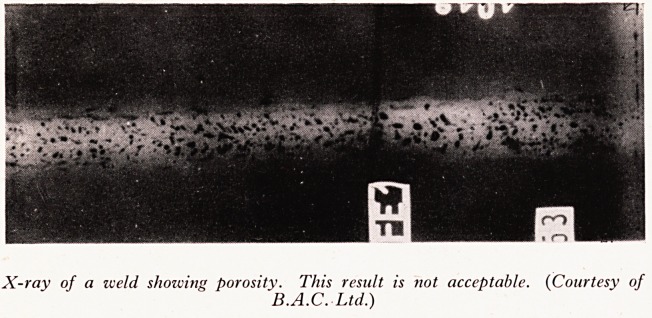


**Figure f5:**